# Impact of using magnifying dental loupes on clinical performance during tooth preparation: A systematic review

**DOI:** 10.4317/jced.61098

**Published:** 2024-02-01

**Authors:** Rayanna-Thayse-Florêncio Costa, Samille-Biasi Miranda, Marcos-Antônio-Japiassú-Resende Montes, Anne-Kaline-Claudino Ribeiro, Adriana-da Fonte-Porto Carreiro, Sandra-Lúcia-Dantas Moraes

**Affiliations:** 1 PhD student. Department of Oral Rehabilitation, Faculty of Dentistry, University of Pernambuco (FOP/UPE). 310 Arnóbio Marques St - Santo Amaro, Recife, Pernambuco, Brazil; 2 MSc student. Department of Dental Materials, Faculty of Dentistry, University of Pernambuco (FOP/UPE). 310 Arnóbio Marques St - Santo Amaro, Recife, Pernambuco, Brazil; 3 Associate Professor of Department of Dental Materials. Faculty of Dentistry, University of Pernambuco (FOP/UPE). 310 Arnóbio Marques St - Santo Amaro, Recife, Pernambuco, Brazil; 4 PhD student. Department of Prosthodontics, Federal University of Rio Grande do Norte. 1787 Salgado Filho St - Lagoa Nova, Natal, Rio Grande no Norte, Brazil; 5 Full Professor. Department of Prosthodontics, Federal University of Rio Grande do Norte. 1787 Salgado Filho St - Lagoa Nova, Natal, Rio Grande no Norte, Brazil; 6 Associate Professor. Department of Oral Rehabilitation, Faculty of Dentistry, University of Pernambuco (FOP/UPE). 310 Arnóbio Marques St., Santo Amaro, Recife, Pernambuco, Brazil

## Abstract

**Background:**

To assess whether using magnification loupes affects tooth preparation working posture performed by undergraduate students and dentistry professionals and whether it influences the quality of the preparation, operator satisfaction and procedure time.

**Material and Methods:**

This review was performed according to the Preferred Reporting Items for Systematic Reviews and Meta-Analyses (PRISMA) statement and registered in the International Prospective Register of Systematic Review (CRD42023482377). Electronic searches were conducted in PubMed/Medline, Cochrane Library, Web of Science, and Scopus databases for relevant articles published up to August 2023. Clinical or laboratory studies evaluating cavities or dental preparations performed with and without magnification loupes were considered eligible. The outcomes were operator working posture, dental preparation quality, operator satisfaction, and procedure time. The quality of the studies was evaluated using the JBI Critical Appraisal tools for Quasi-Experimental Studies.

**Results:**

The searches retrieved 1493 articles. Based on the eligibility criteria, 11 laboratory studies were included, where 410 undergraduate and graduate dental students conducted dental preparations in 1851 dental specimens. Of the 11 selected studies, 6 evaluated the working posture, 6 assessed the quality of the dental preparations, 5 reported operator satisfaction, and 2 assessed procedure time. The results showed that magnifying loupes significantly improved operator working posture, but did not influence the quality of tooth preparations. Although satisfaction reports about experiences with magnifying loupes were favorable, no significant difference was found.

**Conclusions:**

Magnification loupes improved operator working posture. However, clinical studies with more scientific evidence are needed for steady conclusions regarding operator satisfaction and procedural time.

** Key words:**Magnification, dental loupes, tooth preparation, cavity preparation, dental education.

## Introduction

Recent advancements in Restorative Dentistry have led clinicians to perform predictable procedures with clinical applicability of aesthetic and biomimetic concepts requiring shorter chairside time and providing higher success rates (1). Currently, minimally invasive dental preparations have been recommended because reducing or maintaining tooth structure directly affect patient adherence to treatment and clinical longevity (2). Thus, using modern optical magnifying devices associated with suiTable lighting systems in the operative field may help achieve such conditions (3).

Magnification systems range from magnifying loupes to operating microscopes. Thus, it is possible to grade magnifying loupes using a magnification method, including simple, Galilean, and Keplerian loupes, differing from each other in the type and positioning of lenses. Simple loupes have a pair of positive meniscus lenses positioned side by side, Galilean loupes have a system of concave ocular lenses and convex objective lenses, with a conical shape. Keplerian loupes have a cylindrical shape, and due to their complex internal system of convex lenses and prisms, they are longer (4). When compared to simple loupes, binocular loupes (Galilean and Keplerian) have lenses that allow lower angulation for procedure visualization, resulting in an appropriate working distance, regardless of the operator’s experience (5).

Therefore, magnification can be used at any time regardless of professional skill and ability. However, people who require magnifying loupes are recommended to undergo professional training and be advised about their use and benefits (5). During restorative pre-clinical teaching, students are still inexperienced and endure a small operative field that requires high-precision movements. Thus, these devices are advantageous because they improve visual skills and hand-eye coordination, enabling the development of spatial awareness and exposure to more details of dental cavities (6). As result, they could leave out the ergonomic principles promoting better visualization of the teeth (7).

Using loupes is associated with improved visual acuity during procedures without impairing posture (8). Studies show that magnification with loupes are beneficial for professional health and final treatment quality (9-12). This enhanced magnification ensures that workflow is performed with high accuracy and precise control (3). Moreover, it provides better visibility during restorative treatments, ensuring greater ease in detecting restoration ledges, removal of excess composite resin, and better finishing of the margins, allowing minimal wear during dental preparation (13).

Visual acuity during the clinical procedure and an ergonomic working posture are essential for high productivity levels and good quality of life among professionals (7). Thus, this review aimed to assess whether the use of magnification loupes affect tooth preparation working posture performed by undergraduate students and dentistry professionals, and its influence on the quality of the preparation, operator satisfaction, and procedure time. The null hypothesis was that magnification loupes do not affect working posture and that there is no influence on the quality of dental preparations, operator satisfaction, or procedure time.

## Material and Methods

-Protocol

This systematic review followed the PRISMA checklist (Preferred Reporting Items for Systematic Reviews and Meta-analyses) (14) and registered in the International Prospective Register of Systematic Review (CRD42023482377).

-Eligibility criteria

The inclusion criteria in this review was based on the Population-Intervention-Comparison-Outcomes (PICO) strategy: (P)opulation - dentists or undergraduate students; (I)ntervention - dental cavity or teeth preparation using magnifying loupes; (C)omparison - dental cavity or teeth preparation without magnifying loupes; and (O)utcome - working posture. Secondary outcomes were quality of dental preparation, operator satisfaction, and procedure time. Therefore, the research question was as follows: “Does the use of magnification loupes improve operator working posture during tooth preparation?”.

This systematic review adopted the following inclusion criteria: 1) clinical studies (randomized clinical trials, non-randomized clinical trials, prospective studies), 2) sample field of studies in patients or *in vitro* with specimens, 3) studies comparing dental and/or cavity preparations performed with and without magnification loupes, 4) studies that evaluated the quality of dental preparations, 5) studies that evaluated operator satisfaction with loupe use, 6) studies that evaluated operator working posture, and 7) studies that evaluated tooth preparation time with and without using magnification loupes. The exclusion criteria were as follows: 1) clinical studies of the type of case report or case series, retrospective studies, qualitative research about experience reports, and overviews; 2) studies that used the microscope as an assessment tool; 3) studies that analyzed geometric shapes as an assessment method; 4) studies that applied only questionnaires as an evaluation method; 5) electronic surveys; 6) studies focusing on pediatric patients; and 7) dentistry or prosthodontics retreatments.

-Information sources and search strategy

The electronic databases used in this review included PubMed/MEDLINE, Embase, Cochrane Library, Web of Science, Scopus, and https://www.clinicaltrials.com (non-peer-reviewed literature). Two researchers (SBM and RTFC) conducted the search using a combination of specific terms and keywords pooled using the AND and OR Boolean operators to determine all relevant studies. The following search strategy was used: (Magnifying OR Magnification OR Device) AND (Lenses OR Lens OR Loupes) AND (Tooth Preparation OR Dental OR Cavity Preparation OR Prosthodontics OR Dental Porcelain OR Dental Veneers OR Dental Restoration Permanent OR Dentistry OR dental Prosthesis OR Dental Crown).

References of the included articles were screened to identify additional eligible studies. Furthermore, manual searches were performed for journals in the dental prosthesis field (Journal of Oral Rehabilitation, Journal of Prosthodontics, The Journal of Prosthetic Dentistry and International Journal of Prosthodontics) and dentistry (Operative Dentistry, Journal of Dentistry, Dental Materials, Journal of Adhesive Dentistry and Journal of Esthetic and Restorative Dentistry).

-Article selection process

Two independent evaluators (SBM and RTFC) conducted the search and no filter was applied for publication year or language. After searching each database, duplicate studies were removed using the EndNote software (https://endnote.com). All studies retrieved using the search strategy were selected to read the titles and abstracts. Based on the eligibility criteria, a full-text reading was performed, and a third reviewer (SLDM) analyzed the inclusion and selection processes of the other two reviewers. Any disagreements between the reviewers were resolved through discussion. The article selection process is presented as a diagram. Mendeley software was used as the reference manager (Elsevier, Mendeley).

The Kappa Score was used to assess the level of agreement between the evaluators for the inclusion of the studies. The calculation was performed for each electronic database.

-Data extraction and collection process

Data were manually collected from articles by a single reviewer (SBM). The second reviewer (RTFC) conducted the review. Any disagreements between the two reviewers were clarified through a discussion with a third reviewer (SLDM). Data from the included studies were tabulated and interpreted using a standardized Excel spreadsheet (Microsoft, Redmond, WA, USA).

The variables collected from the studies included author, year of article publication, type of study, study groups, evaluation unit, sample size per group, dental preparation procedure performed, characteristics of the operators, evaluation method for operator posture, method, quality assessment method, operator satisfaction assessment method, average procedure time per group, and results. Tables  Table 1,  Table 1 cont.,  Table 2,  Table 2 cont., and  Table 3 present the characteristics of the interest.

**Table 1:**
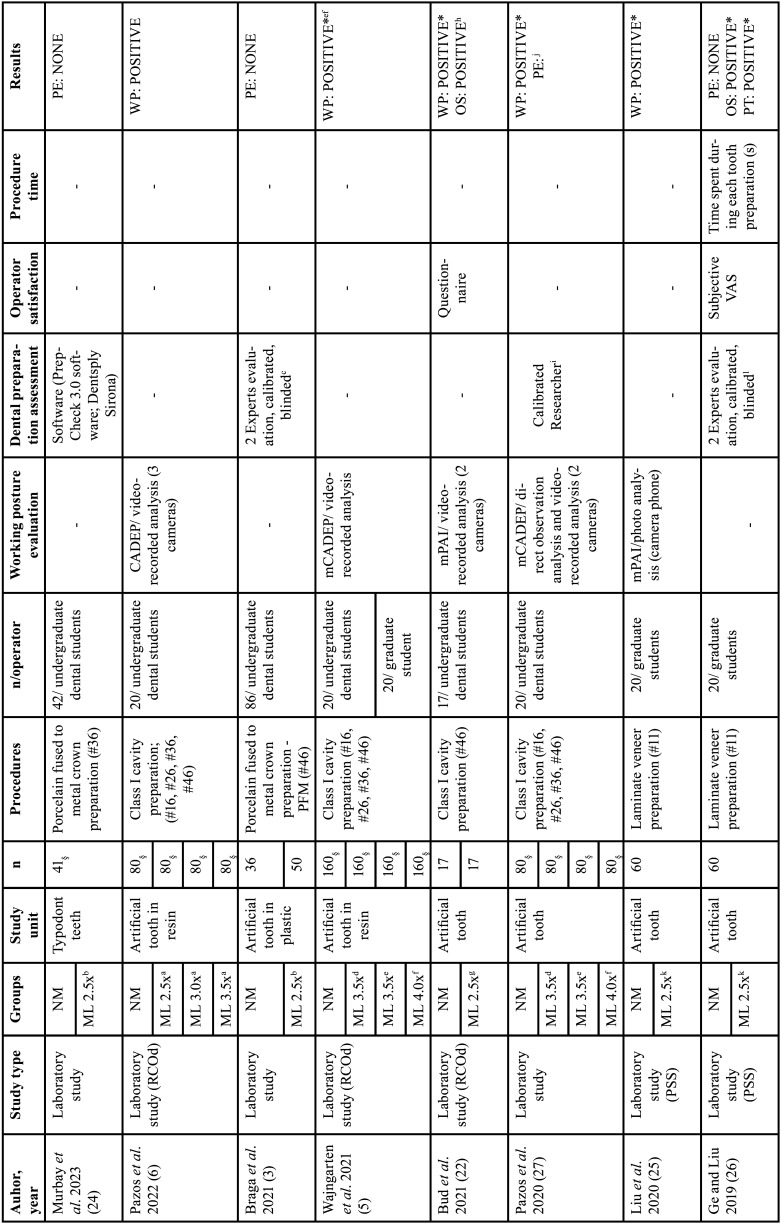
Characteristics of the included studies.

**Table 1 cont.**
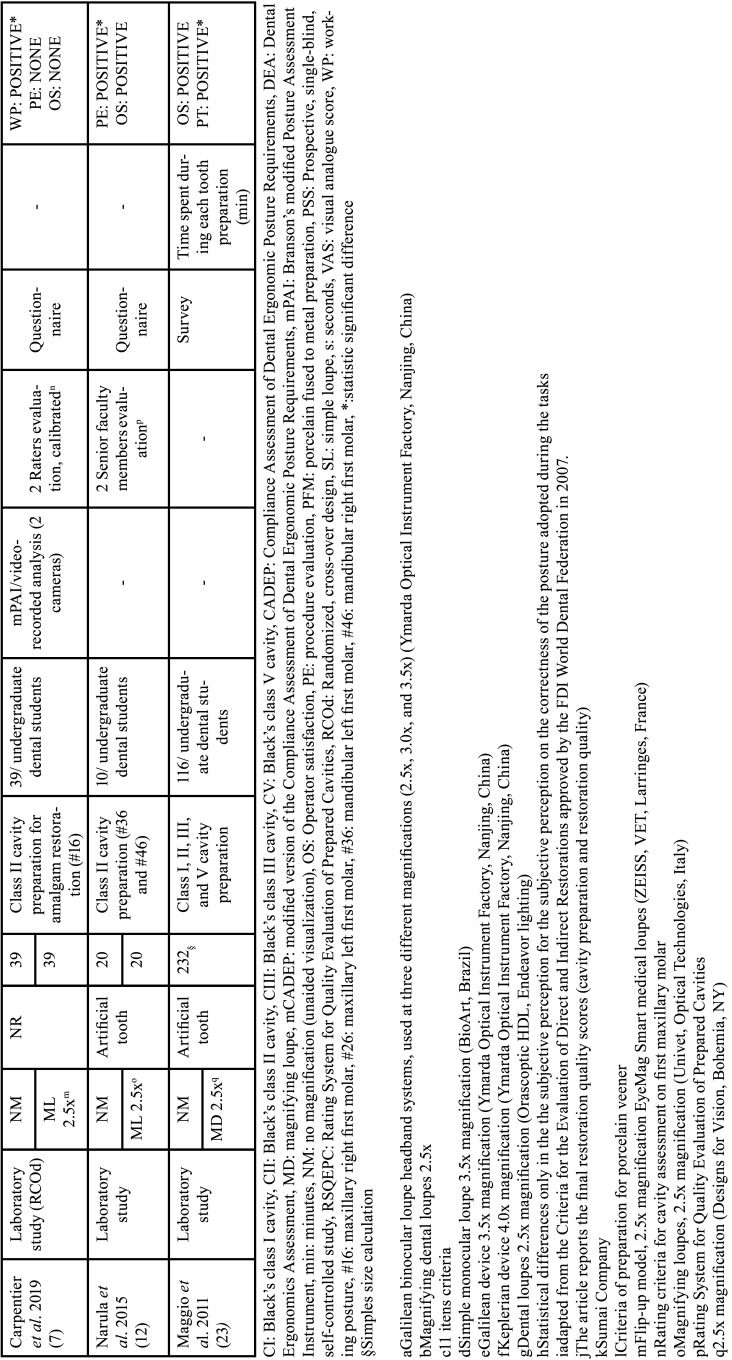
Characteristics of the included studies.

**Table 2:**
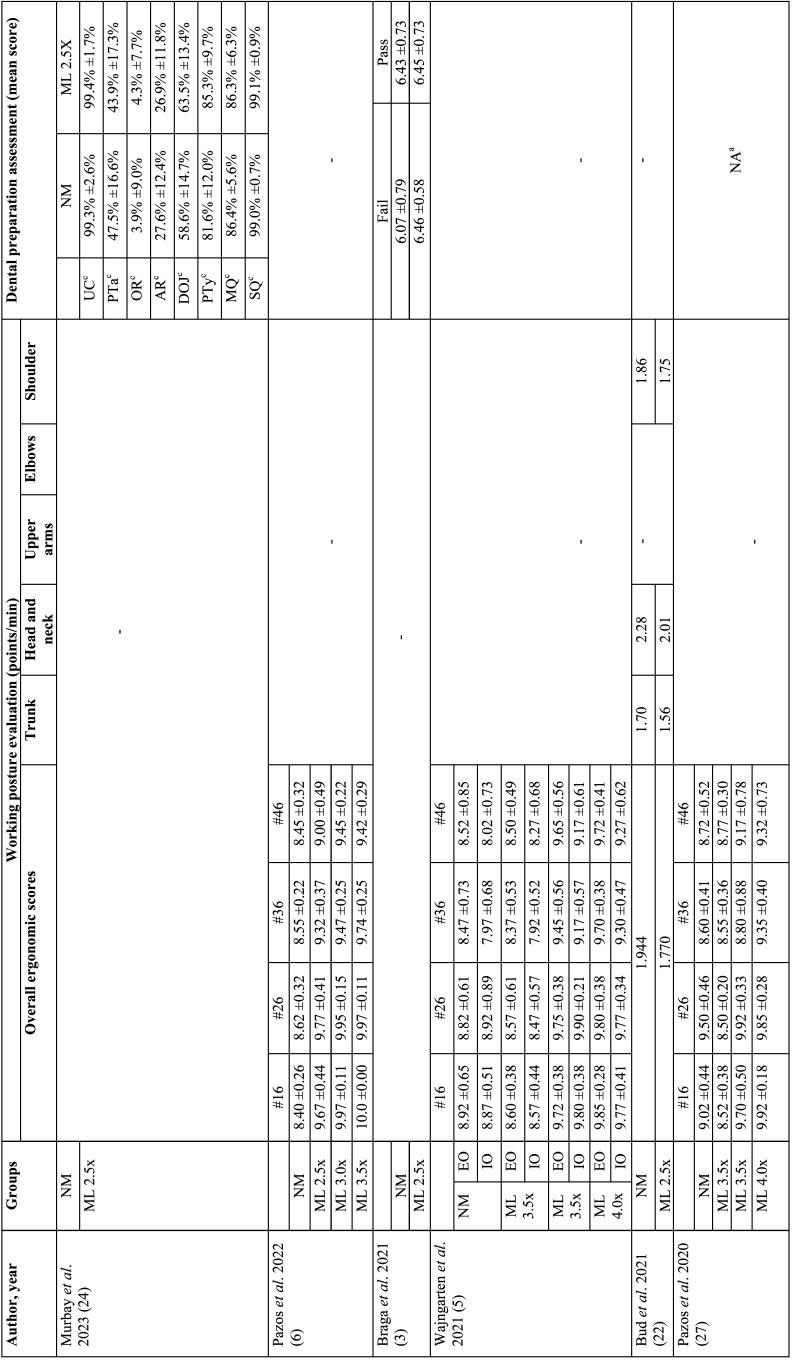
Quantitative data from the included studies (working posture and dental preparation assessment).

**Table 2 cont.**
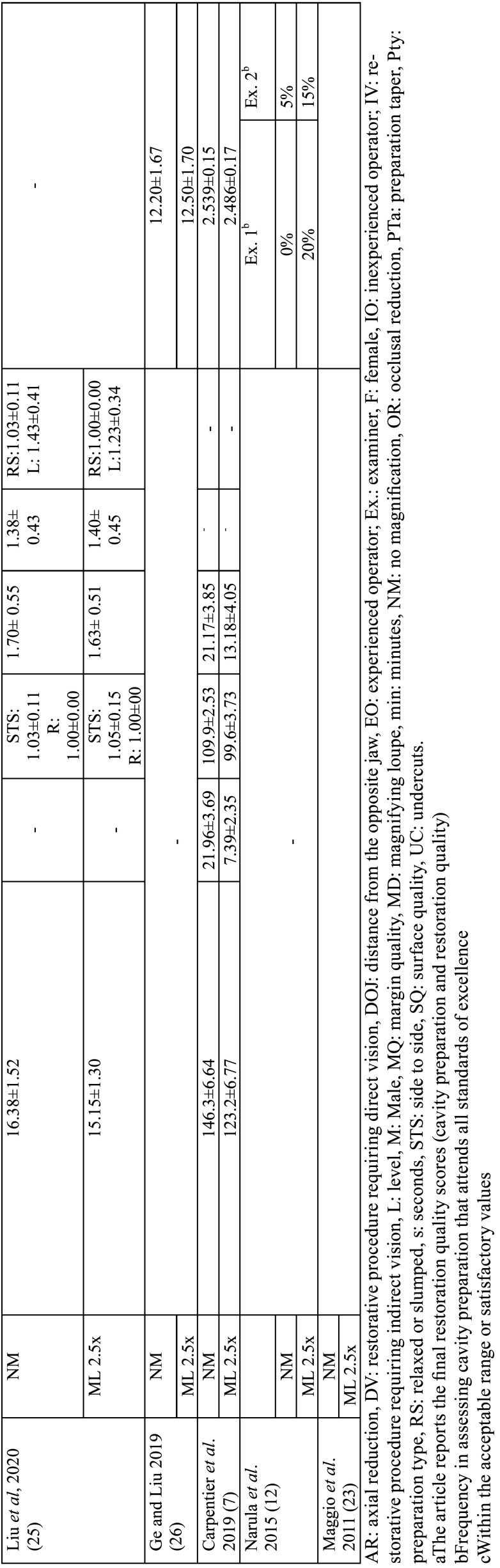
Quantitative data from the included studies (working posture and dental preparation assessment).

**Table 3:**
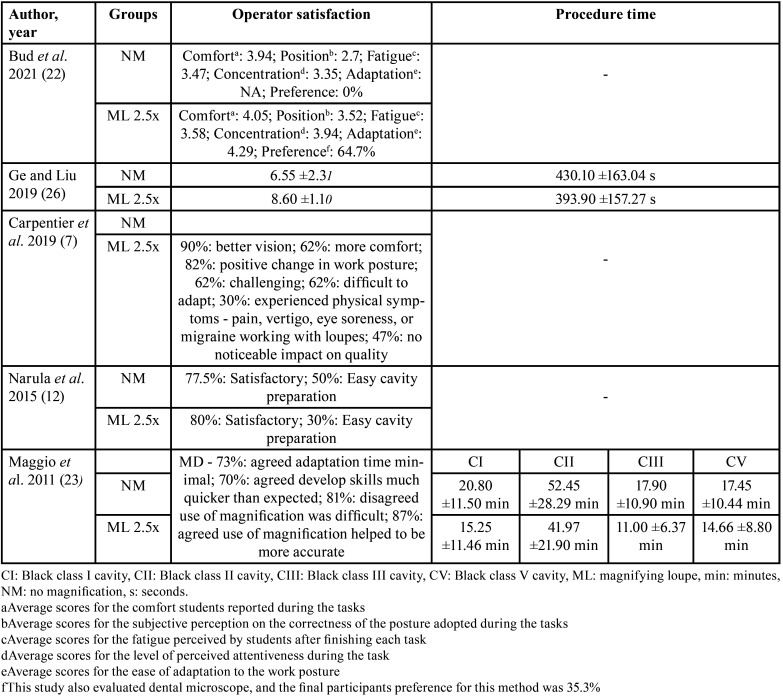
Quantitative data from the included studies (operator satisfaction and procedure time).

-Risk of bias assessment and reporting

The risk of bias in each study was analyzed using the JBI Critical Appraisal Checklist for Quasi-Experimental Studies (nonrandomized experimental studies) (15). This provided a critical analysis of the methodological quality of the studies. Each study was evaluated individually, and JBI provided ten items that were selected based on the characteristics of studies in which the answers were “Yes,” “No,” “Unclear,” or “Not applicable.” Two examiners (SBM and RTFC) conducted the analyses. After these parameters were collected, the studies were classified as having a high (one to three parameters reported), moderate (four to five parameters reported), or low (six or seven parameters reported) risk of bias.

## Results

-Study selection

**Figure 1:**
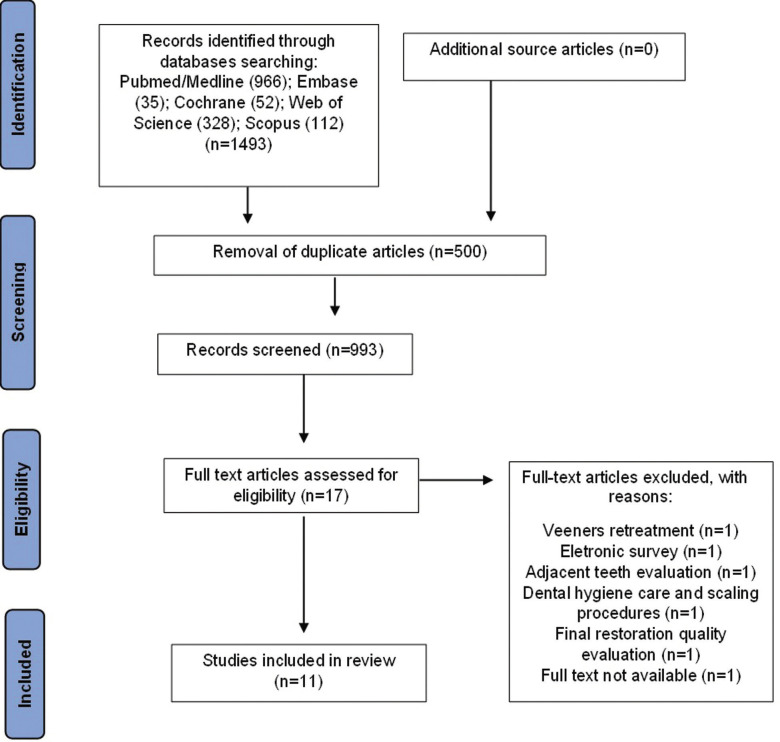
Preferred Reporting Items for Systematic reviews and Meta-Analyses (PRISMA) flow diagram of study selection.

The electronic search of the databases cited in the methodology was performed in August 2023, providing 1493 articles: PubMed/Medline (966) Embase (35), The Cochrane Library (52), Web of Science (328), and Scopus (112). After removing duplicates, 993 samples were retained. Non-peer-reviewed literature (https://www.clinicaltrials.com) returned one record that is ineligible for inclusion. Titles and abstracts were read, and the inclusion criteria were applied, resulting in 17 articles that were potentially eligible for full analysis. The full texts of these articles were read, and six were excluded for the following reasons: veneer retreatment (16), evaluation through electronic research (17), assessment of adjacent teeth (18), full text not evaluable (19), final restoration quality evaluation (20), and dental hygiene care and scaling procedures (21). Thus, 11 (3,5-7,12,22-27) articles were included and extensively reviewed for their approaches and methodologies. Figure 1 shows the details of the article selection process.

The results of the inter-examiner agreement test showed an “almost perfect agreement” between the examiners in the article selection phase. The indices of the databases were PubMed/Medline (0.83), Embase (1.0), Cochrane Library (0.8), Web of Science (1.0), and Scopus (1.0).

-Characteristics of the studies 

Table 1,  Table 1 cont. presents the main characteristics of the 11 studies (3,5-7,12,22-27). All included studies were laboratory experimental studies. All studies were conducted in Dental Universities at the laboratories of pre-clinical disciplines, where 1.851 dental specimens used had artificial teeth (plastic or resin). The comparisons in these studies were as follows: without magnification (control group) and magnifying loupes (intervention groups).

Regarding the operators of the studies, there were a total of 410 participants, divided between undergraduate and graduate dental students. This included 116 students from a study evaluated in Advanced Simulation Course ongoing using technology based on virtual reality (23). In eight studies (3,6,7,12,22-24,27), the undergraduate dental students represented both intervention and control groups. Two other studies (25,26) stated the population as the control group and graduate dental students as the intervention group. In one study (5), the intervention and control groups comprised both undergraduate and graduate dental students (Table 1,  Table 1 cont.).

Regarding dental or cavity preparation procedures, most studies evaluated Black’s Class I cavity preparation (n=4) (5,6,22,27), followed by Class II (n=2) (7,12), porcelain fused to metal preparation (n=2) (3,24), preparation for ceramic laminates (n=2) (25,26), and one study evaluated Black’s classes I, II, III, and V cavities (23) (Table 1,  Table 1 cont.).

Six studies (5-7,22,25,27) assessed working posture during tooth preparation. Methodology for operator posture ranged among the studies, three studies (5,6,27) used the Compliance Assessment of Dental Ergonomic Posture Requirements (CADEP), the other half studies (7,22,25) used Branson’s modified Posture Assessment Instrument. Concerning the evaluation of the quality of dental preparations, assessment of items for four studies were done by two evaluators (3,7,12,26) and one study by one evaluator (27), with variations in the evaluation rating criteria. One study scanned all specimens and evaluated them using the default software of the system (PrepCheck 3.0 software; Dentsply Sirona) (24) (Table 2,  Table 2 cont.).

Five studies (7,12,22,23,26) evaluated operator satisfaction after using magnification loupes; In general, the evaluation methods used were subjective questionnaires where students could report their experiences. Good experiences were reported, such as better vision, more comfort, and easier cavity preparation, one study (26) presented in quantitative scores, 6.55 ±2.31 to unaided vision and 8.60 ±1.10 to magnification group. Unsatisfactory experiences were also reported: not noticeable impact on tooth quality, and physical symptoms (pain and vetigo). Furthermore, only two studies (23,26) analyzed the time required to perform the preparations, and both concluded that using magnifying loupes optimizes the time and speeds up the workflow of the operators. Ge and Liu (2019) reported 430.10 ±163.04s for the unaided group and 393.90 ±157.27 for the 2.5x magnification group. Maggio *et al*. (2011) did similar evaluation, but in four types of Black’s cavities, as shown in Table 3.

-Risk of bias

**Figure 2:**
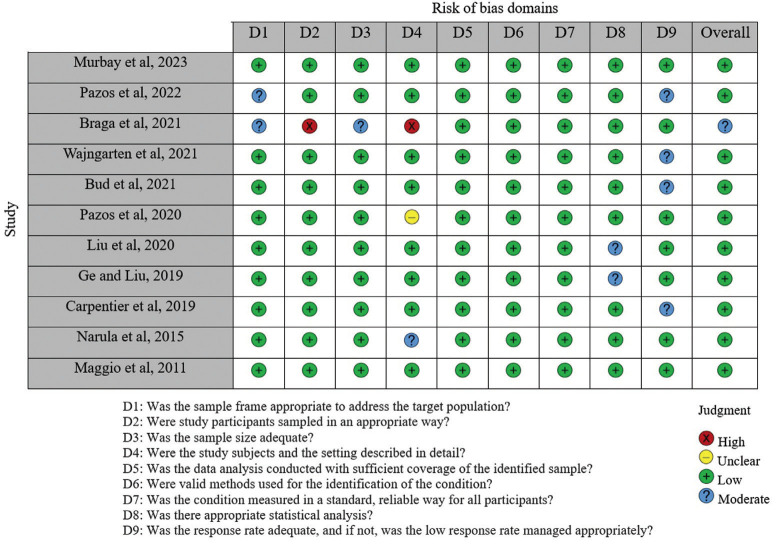
Risk of bias – JBI Critical Appraisal Checklist for Quasi-Experimental Studies (non-randomized experimental studies).

Most of the studies analyzed were of high methodological quality based on the JBI Critical Appraisal Checklist for Quasi-Experimental Studies (non-randomized experimental studies). The risk of bias of the included studies was considered “low” because most items evaluated were categorized as “yes”. Figure 2 presents detailed results of the evaluation.

## Discussion

The study hypothesis that using magnification loupes does not affect working posture was rejected, and the second hypothesis that using magnification loupes does not impact the quality of the procedure was accepted. However, the hypothesis that operator satisfaction and procedure time are similar was considered inconclusive because limited scientific evidence supported this outcome.

Concentration and precision are necessary for dental and/or cavity preparation procedures (28). Moreover, using magnifying loupes provides magnification of the operative field, requiring that the operator remain in a proper back position during work sessions. This keeps them in a good ergonomic position, decreasing the chances of operator movement amendments (22). The results of 6 included studies (5-7,22,25,27) showed that magnifying loupes significantly improved operator posture during dental preparations. These consequences minimizes the risks of diseases associated with work posture, especially musculoskeletal disorders (5), and common diseases among dentists.

The operators included in this study were undergraduate and graduate dental students, and professionals with limited experience in dentistry. Thus, considering the minimal skills, there are reduced trends to watch out for in terms of ergonomics during procedures, promoting frequent tilting of the forward working posture (22). Thus, introducing magnification instruments in undergraduate teaching may be advantageous (3,22,27) reducing the distance between the operator’s eyes and the patient’s oral cavity, and promoting higher comfort for students.

Performing procedures in the dental field requires and depends on a series of factors such as theoretical knowledge, professional experience, and equipment used, highlighting the factors based on the visual tools (12). The reduction of visual acuity is an expected event in the natural aging process (3), mainly after the age of 40 years (29). Therefore, using magnification is advised over the years of dental practice to offset any abnormality in visual capacity, positively impacting this factor (9). Perrin *et al*. (30) reported that magnification promotes a better ergonomic position instead of an improvement in the optical properties of the operator.

Using magnification during tooth preparation, fabrication, impression, and cementation processes of dental crowns and fixed prostheses has been recommended (9). However, findings demonstrated that the unaided vision group had no statistically significant differences from the magnification group on the quality of tooth preparation (3,7,12,24,26,27). One study (31) reported that using magnifying loupes during restoration completion significantly decreased the number of proximal ledges. The possible limitation in improving the quality of dental preparations may be due to the use of magnification by students instead of experienced professionals, thus, these students may not have learned or used loupes previously (32). This learning curve process (13) and the type of magnifying device or degree of magnification could also have an influenced on these results (33).

Regarding operator perception, good experiences were reported while wearing magnification devices, 90% reported better vision while wearing magnification loupes (7), Bul *et al*. (2021) reported that all participants preferred using magnification systems when compared to unaided vision (22). Ge & Liu (2020) reported higher scores for operator satisfaction (8.60 ±1.10) when compared to no use (6.55 ±2.31) (25). However, operators also reported challenges and difficulties with adaptation were also reports from the operators. Carpentier *et al*. (2019) (7) reported 30% of their participants experienced physical discomfort, such as vertigo, migrane, or soreness, while working with loupes.

A study evaluating user’s self-perception after preparations conducted with magnification demonstrated that participants experienced several clinical benefits, including better evaluation of restoration, detection of caries, and greater ease of planning suiTable treatments. Moreover, participants reported an association between dental loupe use in the clinical environment and an improvement in physical appearance, providing greater self-confidence and clinical performance. The results showed an increase in reliability, which could be an important factor because patients connected the use of magnifying loupes to higher professional skills and abilities (10).

However, accessibility in using magnifying loupes is still a major obstacle, since their acquisition and maintenance require a high financial cost (22), which is a difficulty for students in preclinical training and professionals in the first years of dental practice (9). The learning curve is other limitation factor about the magnifying loupes use (13). Some users informed that in addition to being heavy, and making the field of view more restricted and a difficulty in self-positioning during use, causing vertigo, eye soreness, and headache (7,34). In addition to these limiting factors, in some clinical situations, magnification can be harmful by the risk of iatrogenic damage to the enamel during dental preparation, as well as the extension levels > 2.5x significantly reduce the user’s visual ability to detect carious lesions (35). Despite this, the potential long-term benefits may compensate for such limitations (36).

Illuminating the operating field is beneficial for the operator (3,12). Using mounted fiber-optic light on the magnifying loupes is advised by loupe manufacturers because it can enhance light levels, when compared to traditional overhead dental light, up to four times. Light is usually attached to the center of the forehead, closer to the operating field, reducing the probability of shadowing (36). However, the lighting system attached to the magnifying instruments may increase the final cost (1).

Dental students showed good acceptance of magnifying loupes during simulations or clinical training. Thus, the inclusion of these instruments at the beginning of dental education help justify the importance of ergonomics in providing oral care (5,9,22,23). Furthermore, loupes are tools that improve posture because they play a role on reducing distance and improving magnification, reducing musculoskeletal problems in the diary practice and professional career (7). Therefore, it is important to encourage students to use magnifying loupes, promoting its ongoing use, and suggesting their inclusion on the visual magnification training in the university curriculum of Dentistry program (10).

Using magnification in dentistry routine is considered an innovative technique in dentistry practice (3). The applicability has gained popularity (12), boosting the sales market of this equipment (23). However, operators are unaware of the real impact of using these devices (1). Therefore, professionals should understand advantages and limitations, purchase costs of the new equipment, and recognize that handling this tool depends on the learning curve and clinical routine skills (3).

The limitations of this systematic review include the sample of young participants with almost no shift in visual acuity, and insufficient clinical studies evaluating magnification. Heterogeneity in the evaluation methods of ergonomics, position of the operator’s neck during the execution of the procedure, operator satisfaction, and quality of the preparation. Thus, a meta-analysis was not feasible. Additionally, only a few studies have clearly stated the need for proper illumination, which is a fundamental part of magnified loupe systems. Using natural teeth in preparation simulations could also be explored to bring the laboratory study closer to clinical reality. In this review, we emphasize the methodological design of studies as an advantageous factor, with detailed descriptions such as randomization, single-bling, and crossover designs. However, only laboratory *in vitro* studies have been performed, and may have limited the results compared to *in vivo* patients.

## Conclusions

This systematic review demonstrated that using magnification loupes, positively affects the operator’s working posture during dental or cavity preparation. There was no difference in the quality of the preparations when magnifying loupes were used. Clinical studies with more scientific evidence are required to reach steady conclusions regarding operator satisfaction and procedural time.
